# Are health workers motivated by income? Job motivation of Cambodian primary health workers implementing performance-based financing

**DOI:** 10.3402/gha.v9.31068

**Published:** 2016-06-17

**Authors:** Keovathanak Khim

**Affiliations:** Public Health Department, University of Health Sciences, Phnom Penh, Cambodia

**Keywords:** job motivation, health workers, performance-based financing, health reform, financial incentives, Cambodia

## Abstract

**Background:**

Financial incentives are widely used in performance-based financing (PBF) schemes, but their contribution to health workers’ incomes and job motivation is poorly understood. Cambodia undertook health sector reform from the middle of 2009 and PBF was employed as a part of the reform process.

**Objective:**

This study examines job motivation for primary health workers (PHWs) under PBF reform in Cambodia and assesses the relationship between job motivation and income.

**Design:**

A cross-sectional self-administered survey was conducted on 266 PHWs, from 54 health centers in the 15 districts involved in the reform. The health workers were asked to report all sources of income from public sector jobs and provide answers to 20 items related to job motivation. Factor analysis was conducted to identify the latent variables of job motivation. Factors associated with motivation were identified through multivariable regression.

**Results:**

PHWs reported multiple sources of income and an average total income of US$190 per month. Financial incentives under the PBF scheme account for 42% of the average total income. PHWs had an index motivation score of 4.9 (on a scale from one to six), suggesting they had generally high job motivation that was related to a sense of community service, respect, and job benefits. Regression analysis indicated that income and the perception of a fair distribution of incentives were both statistically significant in association with higher job motivation scores.

**Conclusions:**

Financial incentives used in the reform formed a significant part of health workers’ income and influenced their job motivation. Improving job motivation requires fixing payment mechanisms and increasing the size of incentives. PBF is more likely to succeed when income, training needs, and the desire for a sense of community service are addressed and institutionalized within the health system.

## Introduction

The poor quality of health services continues to impede progress toward improving both access to and the use of essential public health services in low- and middle-income countries (LMICs) (1). This is aggravated by the inefficient use of scarce resources, such as manpower, medical equipment, and supplies (2). Motivated and well-trained health workers are essential for high-quality service delivery (3). Poor job motivation affects health workers’ performance, service delivery, and health system performance; improved job motivation is needed to overcome these problems.

The job motivation of health workers has many drivers. Motivation can be defined as ‘an individual's degree of willingness to exert and maintain an effort towards organizational goals’ and can be influenced at many levels: the individual, organization, health sector, and the community (4). Workers’ individual goals, self-concepts, expectations, and experiences are important individual-level determinants of motivation. Health workers may be motivated by several factors. They may, for example, be motivated because they have been trained for the job and feel they have the ability to perform well, and/or they expect a return either monetary, such as financial incentives or income, or non-pecuniary, such as trust or reputation. At the organizational level, structures, processes, resources, relationships with peers, and institutional rules may affect motivation and the ability to deliver a service. This includes the sense of belonging and justice within the organization, which may be affected by the way benefits are distributed among workers. A positive sense of justice requires transparent management and communication. Health workers’ motivations are also influenced by community expectations of the services delivered, and their interactions with and feedback from clients.

Job motivation consists of two broad categories: intrinsic and extrinsic motivation (5, 6). Both represent a return from a job. A doctor, for example, may be motivated in doing his or her job because of the opportunities to meet and assist patients, which is intrinsic motivation (7), and/or because he or she is paid to do the job, which is extrinsic motivation. Extrinsic motivation may also be non-financial, such as recognition, commendation, or opportunities for continuing education or training (8–10).

Motivation varies by gender, the type of health worker, training, and job tenure (11). The factors driving motivation change according to the local context (10). There are numerous factors that drive job motivation and performance, which are country-specific. They include financial incentives, career development, and opportunities for training and continuing education. Factors within the working environment, such as resources, facility quality, management and leadership, working relationships, performance appraisal, and communications, also contribute to worker motivation (12–14). Working conditions greatly influence staff motivation and practices regarding quality improvement and patient safety (15). Health sector reforms in any of these areas can affect motivation through changes in organizational culture, resource provision, reporting structures, human resource management, channels of accountability, and interactions with clients and communities (4).

Health workers in developing countries are often underpaid and poorly motivated. Many engage in predatory practices, such as pilfering supplies from health facilities or charging informal fees to gain additional income (16–18). For these reasons, income is an important factor when designing health system interventions (19). Facility revenues are an important source of additional income for staff (20); therefore, sustaining pay levels and consistent service-related payments are essential for staff motivation and morale. Paying to improve performance is based on an assumption that health workers need incentives to perform. However, the current evidence shows that changes in performance are not due to incentives alone, but to a host of factors (21–23). Studies on incentive schemes have failed to examine the entire remuneration system, and gaps remain in the evidence about the impact of complex remuneration systems on the motivation, retention, and performance of health workers (24).

Despite many previous studies, there is a gap in understanding regarding the role of financial incentives in relation to the overall income and job motivation of health workers. This study aims to improve this understanding by investigating performance-based financing (PBF) as part of the post-2009 public health sector reforms in Cambodia. PBF is a provider-payment mechanism that links providers’ payments to their achievement of agreed-upon performance targets (25). This study explores what drives job motivation for primary health workers (PHWs) in Cambodia and assesses whether and by how much job motivation is affected by income and the financial incentives used in the PBF reform.

## Cambodian health system and reform

Cambodia is a country in Southeast Asia with a population of approximately 15 million. Its health system comprises three levels: the central Ministry of Health (MOH) and subsidiary departments, national centers, and hospitals; the provincial; and the district where front-line service delivery takes place in referral hospitals and primary care centers. Cambodia has 88 operational districts, 97 referral hospitals, 1,105 primary health facilities called health centers (HCs), and eight national hospitals (26). Cambodia's public health system is weak and public health services are of poor quality and under-utilized. About one-third of first contacts for health care takes place in public sector facilities (27). Population health indices are poor, with high mortality rates for infants, children under five years, and women, despite huge investments in infrastructure and human resources. The under-performance of health workers is a major problem. Specific problems include low job motivation, partial or even complete absenteeism, informal and facilitation fees, and dual practices (24, 28, 29). Effective management is also missing. There is a lack of effective performance appraisal, feedback, supervision, recognition, and systems for communications and reporting (30). Addressing these problems and thereby restoring trust in public health services has been an objective of Cambodia's successive reforms.

Cambodia has used different models of PBF since the late 1990s with the aim of speeding up improvements and expanding the coverage of public health services. PBF mechanisms take a variety of forms and utilize contracting as a tool to establish a relationship between purchasers and providers (25). Internal and external contracting differs depending on whether the two parties are from the same or independent legal entities (31). The payment of financial incentives is a cornerstone in contracting relationships. Such incentives improve the behavior and performance of providers and managers, especially in developing countries where staff are underpaid and lack motivation (32). Previous studies show that Cambodian PBF schemes have the potential to improve the efficiency of maternal and child health service delivery (33–36), the responsiveness and entrepreneurship of management, and the accountability of providers (37–39).

**Fig. 1:**
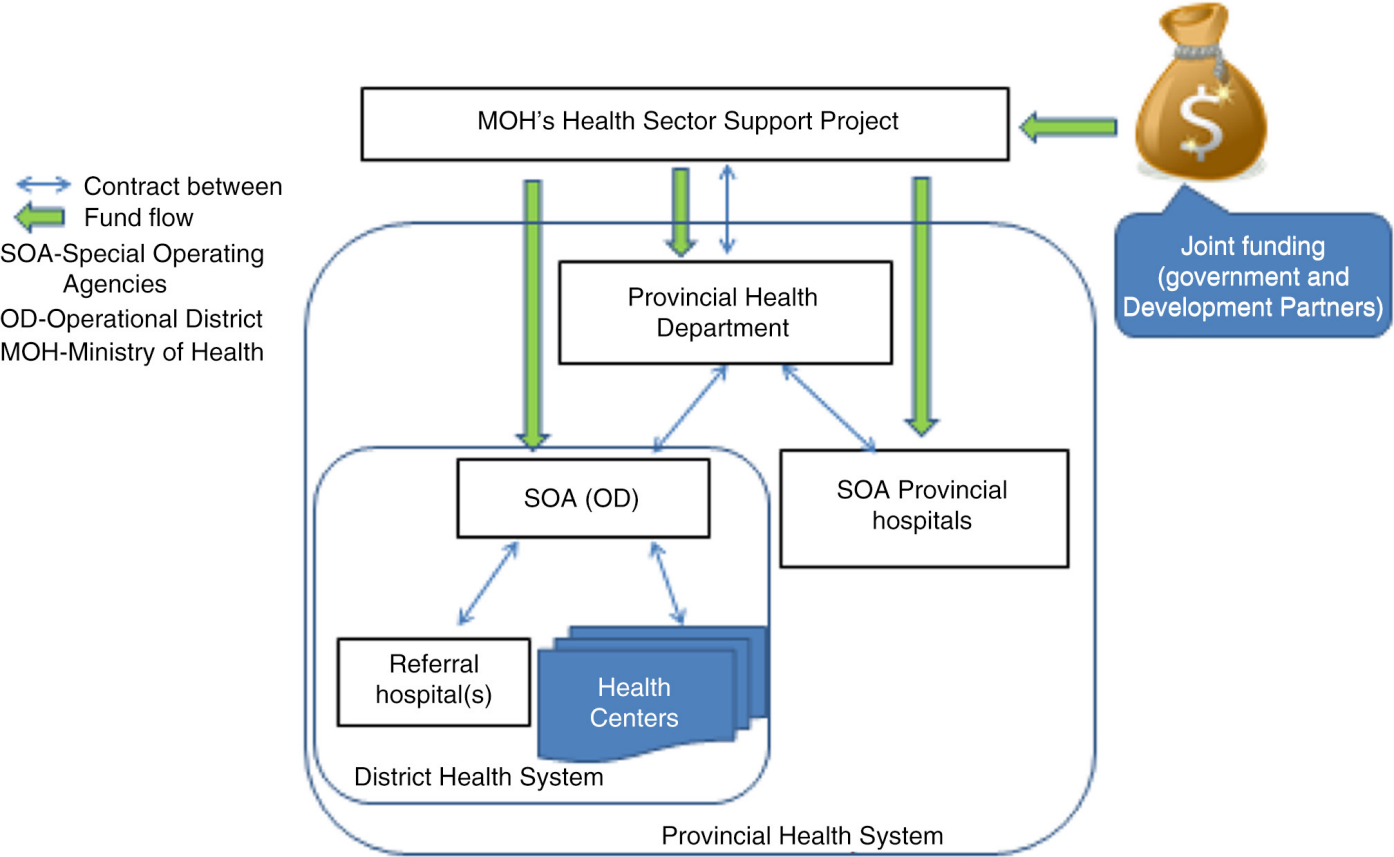
Relationships of units under MOH implementing PBF between 2009 and 2015 in Cambodia. Source: Author.

During 2009, the Cambodian government decided to shift from contracting with non-governmental organizations (NGOs) and instead offer contracts for the management of health districts and provincial hospitals to local government officials. As part of the reform, selected health districts and provincial hospitals became special operating agencies (SOAs) (40), which were provided with specific funding and with greater management autonomy (41). The scheme was piloted and scaled up incrementally from 11 health districts and provincial hospitals in 2009 to 36 in 2015. Through internal contracting, units under the MOH make arrangements based on trust and existing relationships (31). The health districts are contracted with their respective provincial health departments, which are in turn contracted to the MOH. Health workers in the SOAs have performance contracts and are paid incentives according to the achievement of agreed targets (42). Figure 1 shows the contracting relationships within the hierarchies of the Cambodian health system.

## Methods and data

This study seeks to answer the following questions: (1) What is the income of PHWs under the PBF reform? (2) How significant is the financial incentive (SOA incentive) relative to overall income? (3) What influences the job motivation of PHWs in this context? (4) To what extent does income affect job motivation?

### Survey questionnaire development

Data were collected using a cross-sectional survey, informed by a review of the literature and the author's personal experience with the Cambodian health sector. The questionnaire, which included scaled questions regarding motivation, was developed using analyses of health worker motivation in Kenya by Mbindyo et al. (43) and Mutale et al. (11) and the conceptual framework of Franco et al. (4), as a guide. The questionnaire captured respondents’ characteristics, such as gender, age, marital status, level of general education, employment status, job tenure and primary function at the facility, information about their incomes, and their sources and perceptions about the adequacy of job training, and fairness in the allocation of incentives. Twenty items related to job motivation were included in the questionnaire, with a number of items adapted from Mbindyo et al. (43) and Mutale et al. (11), for example, the items ‘I'm satisfied with the opportunities to use my abilities in this job’ and ‘I do this job as it provides long-term security for me’. Further items were formulated to capture different dimensions of motivation, such as appreciation by the community, ability to do the job, and the nature of the job. Respondents were asked to report how much they agreed with the items using scores ranging from one (completely disagree) to six (completely agree). The questionnaire was field tested with 32 PHWs working in HCs not selected for the survey and validated two weeks before the actual data collection began.

### Sampling of primary health facilities and survey participants

Systematic sampling was employed to select 54 HCs from 212 HCs. This was based on an estimate of five PHWs per HC, located in all the 15 SOA districts involved in the reform at the time. The survey was self-administered to a sample of 266 PHWs selected from these 54 HCs. All PHWs at the selected HCs at the time the survey was conducted were approached to be participants.

### Survey implementation

Three Cambodian field assistants were recruited to assist in the data collection for distributing questionnaires, monitoring their completion, and collecting the completed questionnaires under the author's supervision. All providers gave written consent prior to participating in the survey. Data were collected between December 2010 and January 2011, at which point the reform had been implemented for approximately 18 months. The study was part of the author's Ph.D. research and was approved by the Ethics Committee for Health Research of MOH, Cambodia, and by the Institutional Review Board of the University of Melbourne, Australia.

### Statistical analyses

Descriptive statistics (frequencies and means) were generated relating to demography, occupational aspects, sources and levels of income, and perceptions of fairness in incentive distribution. Mean scores of the 20 items were produced and the reliability of the scaled items was checked using Cronbach's alpha coefficient. Missing data were omitted. In order to identify the dimensions of motivation, principal component analysis was conducted. A motivation index score was produced and used as the dependent variable in a multivariable regression model. The model aimed to assess the relationship between income and job motivation. Independent variables were income, the number of members of staff, the level of job training, perceptions of fairness in the allocation of incentives, and respondents’ characteristics (gender, age and general education). Inclusion of these variables, except for respondents’ characteristics, was based on theoretical and statistical considerations. Paying financial incentives, training staff, and hiring additional staff for the health facilities are feasible actions that influence the result of the PBF implementation. Bivariate correlations were performed to check the relationships between the variables. The assumptions of linear regression were checked. The sample size of over 200 was appropriate for the number of variables in the model. The values of tolerance and variance inflation factor indicated that multicollinearity was not a concern. The residual plot showed normality. The Cook's distance, leverage, and studentized residuals showed no influential cases (44, 45). The multivariable model was fitted with SPSS version 20. All significance tests were two-tailed and statistical significance was defined as *p<*0.05.

## Results

### Respondents’ characteristics, job training, and roles at the facilities

The survey achieved a response rate of 98%, with 266 PHWs completing the self-administered questionnaire. Respondents were 54% male and 46% female, with an average age of 36.3 years (Table 1). Most finished grade nine of 12 grades of general education. Most (80.5%, *n*=214) were government employees, and the rest were contract or floating staff. On average, they had worked for 9.9 years at the facility, with seven years being the median. Over 90% of respondents reported receiving professional training, with training in nursing and midwifery predominant. Primary and secondary nurses together made up over 60% of PHWs. Fewer than half of the respondents (42%, *n=*111) said they had received adequate training for their job. The primary roles of the health workers were related to maternal and child health, such as pre- and post-natal care, delivery, and immunization.

**Table 1:** Demographic characteristics of Cambodian primary care workers implementing PBF

Variables	Number	% (mean)
Gender	(*N=*266)	
Male	139	54
Female	127	46
General education	(*N=*265)	
Primary education	32	12
Junior high school	90	34
Senior high school	143	54
Mean age (years)	*N=*266	(36.3)
Employment status	(*N=*266)	
Government employee	214	80.5
Floating staff	29	11.0
Contract staff	23	8.5
Basic professional skill training received:	(*N*=266)	
Primary nurse	91	34.2
Secondary nurse	66	25.2
Primary midwife	64	24.1
Secondary midwife	22	8.3
Pharmacy	1	0.4
Medical assistant	2	0.8
Medical doctor	1	0.4
No training	19	7.1
Training for the job	(*N=*253)	
Training was adequate	111	42.1
Training was not adequate	142	57.9
Primary role at the facility:	(*N=*266)	
Birth spacing/ante-natal/post-natal care	41	15.4
Delivery	47	17.7
Outpatient consultation	42	15.8
Immunization	36	13.5
Health center chief	38	14.3
Reception and general assistance	31	11.7
Stock management/dispensary	31	11.7

### Sources and amount of income

Respondents reported that they received inadequate income from their public sector job and had to perform other activities to earn a living (Table 2). Almost all respondents (98%, *n=*259) reported having other livelihood activities besides working at the health facility. Almost one-third of respondents reported that they had private practices, including running a drugstore or a pharmacy; almost all of these respondents were the chiefs of facilities or in charge of immunization. Aside from these activities, rice farming and livestock raising were among the most common livelihoods.

**Table 2:** Reported income, sources, and livelihoods of Cambodian primary health workers

	Number	%
Respondents who reported		
Income was adequate for living (*N=*239)	41	17.2
They perform other livelihood activities (*N=*266)	259	98.0
Other livelihood activities performed (multiple answers):		
Rice farming	103	39.0
Private practices (including running a drugstore and pharmacy)	80	30.1
Livestock farming	52	19.5
Fruit plantation	34	12.1
Vegetable gardening	20	7.5
Run grocery store	8	2.9
Trade of farm products	5	2.0

Respondents reported that they received a variety of allowances and incentives (Table 3): the SOA incentive, midwifery incentive, night-duty payment, government salary, incentives from user fees, and incentives received from NGO involvement, that is, from the Global Fund or NGOs working in the district. The SOA incentive is the financial incentive received as part of the implementation of SOA. The midwifery incentive is provided by the government for delivery of a live birth at a public health facility, US$15 per live birth delivered at the health center or US$10 at a referral hospital. PHWs also receive a payment for being on stand-by overnight at the facility. Of all income sources, the monthly average of the SOA incentives of US$81 was the highest, accounting for 42% of total income. In most cases, the monthly average of the SOA incentives was higher than the monthly average of the government salary of US$54. Primary health staff reported a monthly average of total income of US$190. Not all the surveyed PHWs had multiple income streams. A number of floating staff, hired on an informal basis to assist with chores at the facility, received the lowest pay.

**Table 3:** Income streams of Cambodian primary health workers at public health facilities

	N^a^	Mean	Minimum	Maximum
Total income	266	190.39	9.76	434.15
SOA incentive	248	81.61	4.88	200.00
Government salary	225	54.44	2.44	104.88
Incentive from other health schemes	75	31.86	2.44	170.73
Midwifery incentive	161	27.4	2.44	121.95
User fees	260	25.71	2.44	146.34
Duty station	195	23.91	2.44	121.95

a Not all workers received the same set of income streams.

### Motivation construct

The 20 items for the index of motivation had a Cronbach's alpha of 0.78, suggesting the items were closely related in measuring motivation (Table 4). Thirteen items had a score of five or higher (the maximum being six), suggesting a high degree of agreement with the statements related to job ability, perceived job value by the community and by the worker themselves, job stability, job importance, and peer relations in the job. The highest scores were for item 17 (the job being important for the health of people, 5.65) and item 16 (the job being valuable for the self, 5.46). Item 12 (working at the facility because of income) had the lowest mean (2.98). The 20-item motivation construct had a mean of 4.93 (*n=*258, median 5, standard deviation 0.418, minimum 3.4 and maximum 5.95).

**Table 4:** Mean score for the 20-item motivation construct

		Mean^a^
1	I have the capacity to do my job well.	5.00
2	I need additional training on my job skills.	5.47
3	I believe community people value my job.	5.07
4	I work here because it is the government job that is stable and long lasting.	5.33
5	I like this job because I receive high regard in the community.	5.19
6	I like the income from working at this HC.	4.18
7	Health services of this HC are trusted by community people.	5.08
8	Often, the chief commends my job.	4.49
9	I continue to work here expecting the income will rise.	4.74
10	Normally, I feel happy to come to work at this HC.	5.15
11	I work here because I have received training for this job.	5.09
12	I work here because of the income.	2.98
13	I am satisfied with the opportunities to use my abilities in performing the job.	5.30
14	I am proud of working at this HC.	5.29
15	I do what I have to do without being asked or told to do.	4.26
16	My job here is valuable for me.	5.46
17	My job is important for the health of people.	5.65
18	I continue to work here because there is no other work that is better.	4.79
19	I get along well with other staff.	5.28
20	Staff here trust each other well.	4.97
	Total motivation index score	4.93

a On a scale from one to six, where one is completely disagree and six is completely agree.

All 20 items had a coefficient higher than 0.4, which was used as a cut-off point. Each item had a shared variance of at least 16% with the factor under consideration. Based on these criteria, six latent factors were confirmed from the factor analysis (Table 5). All the latent factors loaded higher than 0.6.

**Table 5:** Six dimensions of job motivation from principle component analysis

	Job benefit	Organizational commitment	Job satisfaction	Job relation	Job nature	Job competence
I like this job because I receive high regard in the community.	0.746					
I believe community people value my job.	0.735					
Health services of this HC are trusted by community people.	0.643					
I like the income from working at this HC.	0.619					
I work here because it is the government job that is stable and long lasting.	0.527					
Normally, I feel happy to come to work at this HC.		0.766				
I am proud of working at this HC.		0.686				
I continue to work here because there is no other work that is better.		0.565				
I need additional training on my job skills.			0.789			
My job is important for the health of people.			0.709			
I am satisfied with the opportunities to use my abilities in performing the job.			0.524			
My job here is valuable for me.			0.500			
I get along well with other staff.				0.672		
Often, the chief commends my job.				0.622		
I continue to work here expecting the income will rise.				0.589		
Staff here trust in each other well.				0.563		
I work here because of the income.					0.725	
I work here because I have received training for this job.					0.581	
I do what I have to do without being asked or told to do.						0.794
I have the capacity to do my job well.						0.523

### Important factors of job motivation

In the bivariate correlations, income, age, and fair incentive allocation had a weak positive correlation with job motivation (correlation coefficient of income 0.180, *p<*0.01; age 0.179, *p<*0.01; fairness 0.172, *p<*0.01). The multivariable model fitted the data well. Age, income, and perceived fairness in incentive allocation each had a statistically significant association with job motivation, while controlling for gender and general education (Table 6). Compared to income and perceived fairness, age had the strongest association with job motivation (standardized coefficient of income 0.143, *p*=0.043; fair incentive allocation 0.156, *p*=0.025; and age 0.187, *p*=0.038). The lower bounds of the coefficients of income and age close to zero suggest that these factors had no effect on job motivation. The results suggest that, with all the other variables remaining fixed, an increase of one unit (US$2.5) in income was associated with an increase of 0.143 in the motivation score; the perception that the SOA incentive was fairly allocated was associated with an increase of 0.156 in the motivation score; an increase of one year in age was associated with an increase of 0.187 in the motivation score. The number of staff at the facility and job training had no association with job motivation (coefficient of number of staff 0.109, *p*=0.115; job training −0.043, *p*=0.531).

**Table 6:** Associations with job motivation in linear regression

	Unstandardized coefficients		Standardized coefficients			95% confidence interval for B	
					
	B	Std. error	Beta	*t*	*p*	Lower bound	Upper bound
**Income**	**0.001**	**0.000**	**0.143**	**2.04**	**0.043**	**0.000**	**0.002**
Job training	−0.036	0.058	−0.043	−0.628	0.531	−0.151	0.078
Number of staff	0.018	0.012	0.109	1.581	0.115	−0.004	0.041
**Fair incentive allocation**	**0.134**	**0.059**	**0.156**	**2.252**	**0.025**	**0.017**	**0.251**
Gender	−0.013	0.059	−0.016	−0.227	0.821	−0.129	0.102
**Age**	**0.008**	**0.004**	**0.187**	**2.089**	**0.038**	**0.000**	**0.015**
General education	0.016	0.014	0.099	1.125	0.262	−0.012	0.043

*N*=212; model adjusted *R*^2^=0.062; ANOVA (*F*=2.981, df=7, *p=*0.005). The bold texts and values are to highlight factors of statistical significance.

## Discussion

### Effect of financial incentive and income

This is the first quantitative study of job motivation of health workers in PBF schemes in Cambodia. It provides important evidence about the relationship between income level and job motivation. The results indicate that a change in income can significantly influence job motivation, but this depends on the unit of income change and the effect on motivation increases with larger increments of change. The SOA incentive is a substantial addition to the meagre formal income of PHWs. Over 80% of PHWs in this study reported that their basic income was not adequate for a decent living and many of them resorted to other livelihoods, including dual practices, to supplement their formal income. The workers reported an average monthly income of US$190, which is well below the living wage. A study conducted in 2009 estimated that the average basic household consumption in Cambodia was approximately US$273 per month in rural areas and US$379 in urban areas other than Phnom Penh (46). This finding supports the evidence from qualitative research that low income is a cause of poor performance among Cambodian public health workers (9, 28, 35).

The findings relating to income reinforce the message that the financial incentives from PBF, which on average accounted for 42% of total income, are essential to supplement health workers’ incomes and help to motivate them in their jobs. A similar finding was reported in PBF schemes elsewhere (21). The predominant use of financial incentives indicates the recognition of the problem of low incomes in developing countries; these incentives have been widely applied in projects and programs supported by development partners in Cambodia (38, 47) and elsewhere and as a rationale for PBF schemes (48). As this study shows, over one-third of respondents reported running a drugstore or private healthcare practice, taking them away from their public sector work. The lack of financial incentives from public sector jobs has been associated with domestic and international migration, leading to health worker shortages in rural areas in Cambodia and many other developing countries 32, (49–52). If not adequately addressed, outside work and other illicit income-generating practices can damage the trust in and the quality of public health services (53, 54).

### The need for streamlining payment mechanism

The multiple sources of income in this study show the complexity of payment systems. Cambodia has been struggling with pay reform, factoring in different development partners’ funded interventions and incentives (55). Health centers are a convergent point of health services and interventions; PHWs bear heavy workloads and have to shift roles frequently to meet different service demands (56). It is crucial to have sufficient systems in place to coordinate interventions, gauge human resources needs, and streamline different payments. Such systems would help to avoid the overlapping and wastage of resources, improve retention of health workers, and improve the sustainability of interventions.

### Fair distribution of benefits matters

Alongside the complexity of different payments is the need for the fair distribution of benefits to health workers. The results above show that the perception of the fair distribution of incentives can affect job motivation. The bigger the incentive payment, the more important it is to have a mechanism to ensure the fair distribution of benefits. This particularly applies to the SOA incentive, which accounts for 40% of their total income. Early research indicates that the perception of fairness in the distribution of benefits helps to maintain strong morale and a spirit of teamwork among workers (13). This sense of fairness is essential in healthcare delivery, which is labor intensive and involves different skills and disciplines; teamwork and cooperation are necessary for the successful delivery of services (57).

### Non-financial factors in job motivation

Attention should equally be paid to non-financial incentives. The results from this study indicate that PHWs reported a high motivation index score (4.93, on a range from one through six) despite reporting low income. The item related to income as a reason for working at the facility scored particularly low (2.98), while other items relating to respect by the community, job value, peer relations, and job training scored very highly. This indicates that, relative to other aspects of the job, income is not the primary purpose of the job as generally thought. There may be two explanations. First, understandably, income would not be a reason to take a job because it is generally known that public sector job incomes are small. The job may bring other benefits that are rewarding, for example, clients for private practice and community recognition. Second, the high scores of items 5, 17, and 19 related to community and relations suggest that the PHWs are altruistic and value community services, and good community and peer relations. This finding was consistent with the findings of previous research on the sense of community service, community respect (58), job nature, job satisfaction (15), and working relations (14). Improving job motivation does not always require monetary investment and mostly involves promoting a sense of community service and belonging, and providing opportunities for training and the development of professional skills (54, 59).

### Capacity development

The study highlights the need for the adequate training of health workers. Almost 60% of Cambodian PHWs reported that their job training was inadequate, as shown by the high score of item 2 (Table 4). This is particularly troubling, as PHWs are the front-line service providers who see patients with diverse health problems (60). This lack of training limits the provision of quality health services and affects health workers’ job satisfaction and motivation. Local managers need to be empowered to negotiate contracts and ensure timely access to training resources (61). Resolving these issues may not only require increased budgets for capacity development, but also a good system for monitoring and analyzing training needs and for coordinating capacity-building activities at national and sub-national levels (62, 63).

This survey was conducted one and one-half years after the initiation of the PBF reform and provided useful information for gauging its implementation and how this has influenced job motivation. The current internal contracting arrangement is being examined and re-configured for another term of implementation. A follow-up survey will be necessary to monitor changes in job motivation and assess the further impact of PBF implementation.

### Limitations

This study has a number of limitations. First, the data were collected from PHWs based on their perceptions of the situation at the time of survey, which may have changed. Second, the level of income and the sources of income may have been under-reported. Third, the data and results from analysis were limited to PHWs in health districts implementing the reform. Nonetheless, the findings on job motivation among these workers are relevant and useful for policy-making to improve PBF in Cambodia.

## Conclusions

This study highlights what underlies the job motivation of public health workers in the context of PBF reform. This knowledge can improve targeting and increase the impact of PBF interventions. PHWs in Cambodia in districts implementing the PBF reform were generally well motivated and placed a high importance on their community, the job and its benefits, and the organization. Improvements in their job motivation must promote the sense of community service, job value, and ability. Immediate policy implications are to ensure that the mechanism for paying financial incentives to health workers is just and objective, and health workers are trained in required skills. In developing countries, PBF as a health system reform method is likely to succeed if the needs of health workers are addressed and integrated as part of comprehensive human resources management.
